# In vitro co-expression chromatin assembly and remodeling platform for plant histone variants

**DOI:** 10.1038/s41598-024-51460-6

**Published:** 2024-01-10

**Authors:** Petra Banko, Kei-ichi Okimune, Szilvia K. Nagy, Akinori Hamasaki, Ryo Morishita, Hitoshi Onouchi, Taichi E. Takasuka

**Affiliations:** 1 Research Faculty of Agriculture, Hokkaido University, Sapporo, 060-8589 Japan; 2 Graduate School of Global Food Resources, Hokkaido University, Sapporo, 060-0809 Japan; 3 Department of Molecular Biology, Institute of Biochemistry and Molecular Biology, Semmelweis University, Budapest, 1094 Hungary; 4 CellFree Sciences Co., Ltd, Matsuyama, 790-8577 Japan

**Keywords:** Epigenetics, Plant molecular biology, Chromatin remodelling, Histone variants

## Abstract

Histone variants play a central role in shaping the chromatin landscape in plants, yet, how their distinct combinations affect nucleosome properties and dynamics is still largely elusive. To address this, we developed a novel chromatin assembly platform for *Arabidopsis thaliana,* using wheat germ cell-free protein expression. Four canonical histones and five reported histone variants were used to assemble twelve *A. thaliana* nucleosome combinations. Seven combinations were successfully reconstituted and confirmed by supercoiling and micrococcal nuclease (MNase) assays. The effect of the remodeling function of the CHR11-DDR4 complex on these seven combinations was evaluated based on the nucleosome repeat length and nucleosome spacing index obtained from the MNase ladders. Overall, the current study provides a novel method to elucidate the formation and function of a diverse range of nucleosomes in plants.

## Introduction

The eukaryotic genome is compacted and organized into chromatin; a long-range nucleoprotein complex made up of repeating units of nucleosomes. A nucleosome consists of a histone octamer, comprising two copies each of canonical histones H2A, H2B, H3, and H4, and approximately 147 bp DNA, which wraps around the octamer in ~ 1.7 turns^1^. In addition to a higher-order DNA packing, chromatin functions in various cellular processes, including transcriptional regulation, replication, DNA repair, and others, through epigenetic modifications of both DNA and histones and the incorporation of histone variants into nucleosomes^2–4^. The deposition of histone variants conveys unique properties to nucleosomes that affect chromatin stability^5^, selectivity to epigenetic enzymes^6,7^, and higher-order chromatin structure^8,9^, which in turn, greatly diversifies the chromatin landscape.

In *Arabidopsis thaliana*, there are four types of histone H2As, including the canonical H2A, and three variants, H2A.X, H2A.W, and H2A.Z. Variants differ from canonical H2A by the length or unique motifs of their C-terminal tails, and conserved residues in their histone fold domains^10,11^. H2A.X is known to play a critical role in signaling DNA damage response through C-terminal phosphorylation^3,12,13^. H2A.Z is reported to be mainly associated with transcriptional regulation in plants^14,15^ and other eukaryotes including humans^16^ and yeast^17^. H2A.W is a plant-specific histone variant, found almost exclusively in heterochromatin^8^ and acts in DNA damage response in these highly condensed regions^3^. *Arabidopsis* H2Bs are classified into three groups based on their amino acid sequences and expression patterns^18^. Class I H2Bs, wherein H2Bs with canonical histone-like features are categorized, mainly function in somatic tissues, and show peak expression levels in the actively dividing cells^18,19^. Class II/III H2Bs are known to control chromatin structure in the reproductive tissues^18,20^. However, our current understanding of physiological functions of H2B variants in plants is still limited. Three main histone H3s are known in *A. thaliana*, including the canonical H3.1 and the variants H3.3 and CENH3, all of which are known to incorporate into different regions of the genome^21,22^. The functions of H3.3 and centromeric H3 are well-conserved across eukaryotes, in which H3.3 has been linked to transcriptional activation^23^, and the centromeric H3 is reported to aid in chromosome segregation through directing kinetochore assembly^24,25^. In contrast, there is no histone H4 variant in *A. thaliana*. The histone variants used in this study and their reported characteristics are listed in Table 1.

**Table 1:** Summary of histone variants in *A. thaliana* used in this study and reported functions.

Core histone	Variant type	Genes name	NCBI RefSeq	Characteristics	References
H2A	H2A	HTA10	NM_103984.4	S-phase linked expression, uniformly marks gene bodies excluded from heterochromatin	^8^
	H2A.X	HTA3	NM_104344.2	abundant in chromatin, involved in DNA repair in euchromatin	^3,8^
	H2A.W	HTA6	NM_125380.4	localizes in heterochromatin, enhances chromatin condensation through a higher propensity to promote fiber-to-fiber interactions, maintains transposon silencing	^3,8,26^
	H2A.Z	HTA9	NM_104152.4	enriched at the TSS of expressed genes, enriched in gene bodies of response genes	^8^
H2B	Class I	HTB9	NM_114467.4	mostly localize to gene bodies and mainly expressed in somatic tissues	^18^
H3	H3.1	HTR2	NM_100790.3	S-phase linked expression, enriched in silent areas of the genome, and associated with heterochromatin marks	^21^
	H3.3	HTR5	NM_001342565.1	predominantly localized towards the 3’ end of genes and is positively correlated with gene expression, excluded from heterochromatin, all genes code for identical proteins, suggested to have a role in post embryonic development in seed	^21,27^
	CENH3	HTR12	NM_001331269.1	marks centromeres, essential in kinetochore formation	^25,28^
H4	H4	HIS4	NM_128434.4	S-phase linked expression, uniformly marks gene bodies	

In plants and other eukaryotes, histone variants may form over hundreds of different types of nucleosomes^2^. For instance, the unstable H2A.Z and H3.3 double variant-containing nucleosomes are specifically enriched at the promoter and enhancer regions, implying their roles in promoting gene expression in humans^29^. To our knowledge, however, the physiological functions of such combinations have been mostly elusive in plants except for few cases. For instance, it was shown that H2A variants form homotypic nucleosomes, consisting of two copies of H2As from the same variant types in *A. thaliana*^5^. On the other hand, H3.1 and H3.3 were shown to be present in both hetero- and homotypic nucleosomes^30^. However, the physiological roles of neither homotypic or heterotypic enriched region has not been determined. To date, how the above-mentioned histone variant-containing nucleosomes affect the function of remodeling or histone chaperone activity should be further assessed biochemically.

To this end, we attempted to reconstitute twelve *A. thaliana* nucleosome combinations from canonical and variant histones using the wheat germ cell-free co-expression chromatin assembly system described in recent studies^31–33^. Among the twelve tested combinations, seven types of nucleosomes were successfully reconstituted. Furthermore, we aimed to remodel these seven types of variant-containing nucleosomes using an ISWI-type remodeling complex, comprising CHR11 and DDR4^34,35^.

## Results and discussion

### Cell-free synthesis of canonical histones and variants

**Figure 1:**
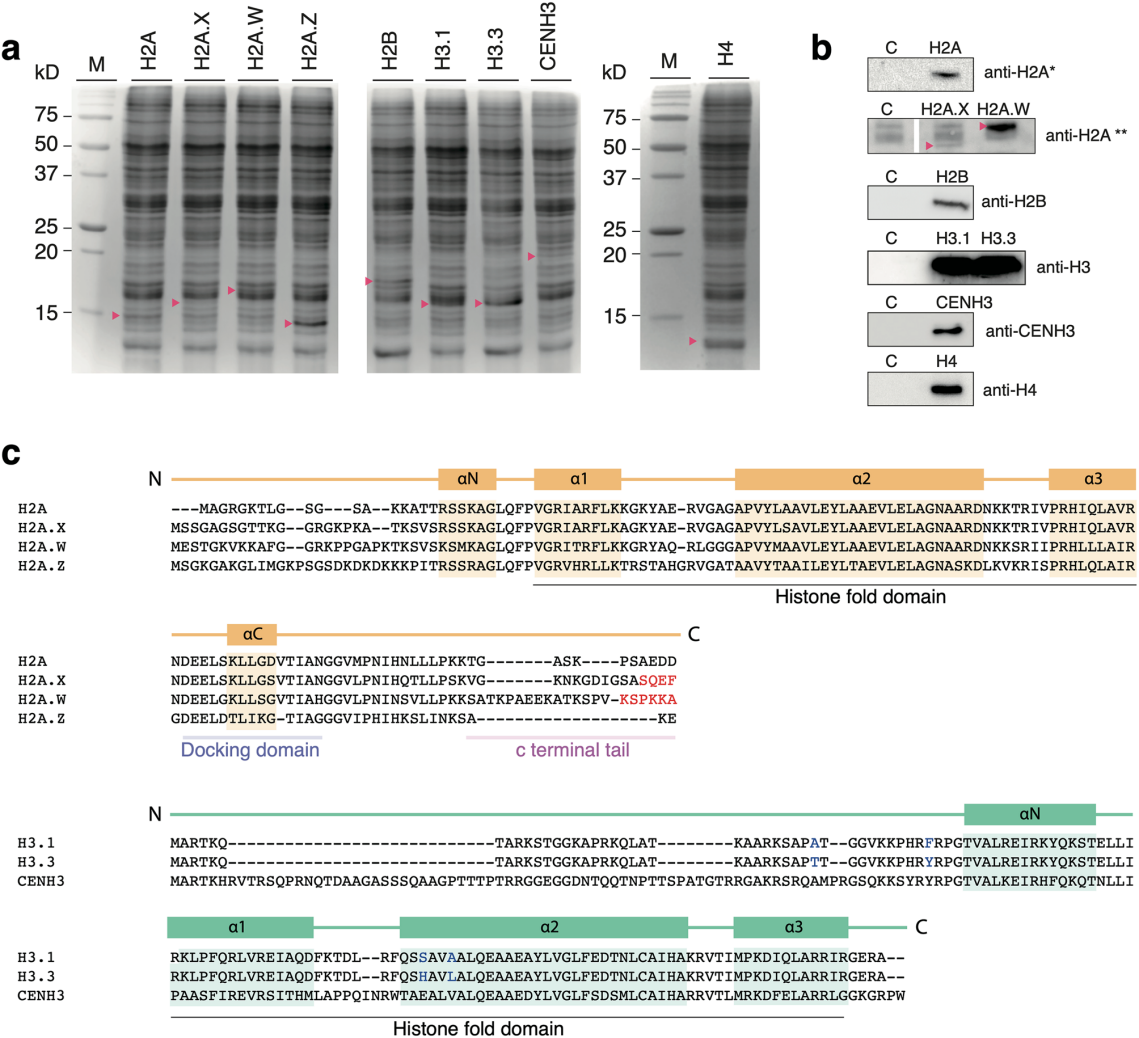
Canonical and variant histones synthesized by wheat germ cell-free synthesis. (**a**) *A. thaliana* canonical histones, H2A, H2B, H3.1, and H4, and histone variants, H2A.X, H2A.W, H2A.Z, H3.3, and CENH3 were cell-free synthesized, and products were analyzed on an 18% SDS-PAGE followed by CBB staining. The red-filled triangles indicate the histone proteins in the wheat germ endogenous protein background. M: protein molecular weight marker. (**b**) Histone-specific antibodies, anti-H2A, anti-H2B, anti-H3, anti-CENH3, and anti-H4, were used to analyze unpurified histone products along with the wheat germ extract background (denoted by C) by western blot analysis. Two types of antibodies were used, for the detection of the canonical H2A (*H2A specific antibody), and H2A.X and H2.W variants (**H2A acidic patch-specific antibody). H2A specific antibody recognizes histones H2A, H2A.X, H2A.W, but not H2A.Z. Note that the H2A acidic patch-specific antibody interacts with the endogenous wheat germ protein background, here red-filled starts indicate the bands corresponding to H2A.X and H2A.W, both of which were detected only when either H2.X or H2A.W was expressed. Uncropped blots are available in the Supplementary Information (S1). (**c**) Multiple sequence alignments of canonical histone H2A and H3.1 along with their respective variants, H2A.X, H2A.W, H2A.Z and H3.3, CENH3, used in this study are shown. The structural features of histone variants are based on HistoneDB 2.0 database and annotated above the alignments. Alpha helices within the alignments of H2A and H3 variants are highlighted in orange and green, respectively. The characteristic C-terminal tail motifs distinguishing the H2A.X and H2A.W variants are indicated with red-colored fonts, and the four characteristic amino acid substitutions between H3.1 and H3.3 are indicated with blue-colored fonts.

Each *A. thaliana* canonical histone, H2A, H2B, H3.1, and H4, and their variants, including three H2A variants, H2A.X, H2A.W, and H2A.Z, two H3 variants, H3.3 and CENH3 were cell-free synthesized, and all proteins were analyzed by SDS-PAGE and western blotting. Histones, including H2A, H2A.W, H2.A.Z, H2B, H3.1, H3.3, CENH3, and H4, were determined to be expressed at their expected molecular weights, but few products were not easily visible, partly due to overlapping wheat endogenous protein bands (Fig. 1a). In Fig. 1b, western blotting was conducted using corresponding anti-histone antibodies, and two different anti-H2A antibodies were used to detect the canonical H2A and H2A.X and H2A.W variants. The anti-H2A-specific antibody detected the synthesized canonical H2A, while this antibody did not detect the synthesized H2A.X nor H2A.W. To note, this result also provided evidence that there is no wheat endogenous H2A in the wheat extract (S1). Thus, H2A.X and H2.W variants were detected by an anti-H2A acidic patch antibody. This antibody exhibits low specificity to histone H2A species, and the additional proteins detected in the background are unlikely derivatives of wheat histones. Overall, all histone products used in this study were confirmed by western blotting except for H2A.Z, which was not detected by commercially available antibodies. Instead, we determined the expressed H2A.Z by LC–MS/MS with ~ 75% coverage (Supplementary Method and Supplementary Table 2). Whilst, individual H2B, H3.1, H3.3, CENH3, and H4 were recognized by the corresponding antibody, and we concluded that besides H2A, there are no other histones present in the wheat extract.

The different molecular weights determined among canonical and variant H2As were consistent with their amino acid sequences (Fig. 1c). Between H3.1 and H3.3, there are only four amino acid alterations, two in the N-terminal non-structural region and two in the α2 helix (Fig. 1c). While CENH3 has a large insertion of ~ 38 amino acids in its N-terminal tail, compared to H3.1, which is suggested to direct CENH3 loading into the centromeres of meiotic chromosomes^25^.

### Reconstitution of histone variant-containing chromatin

**Figure 2:**
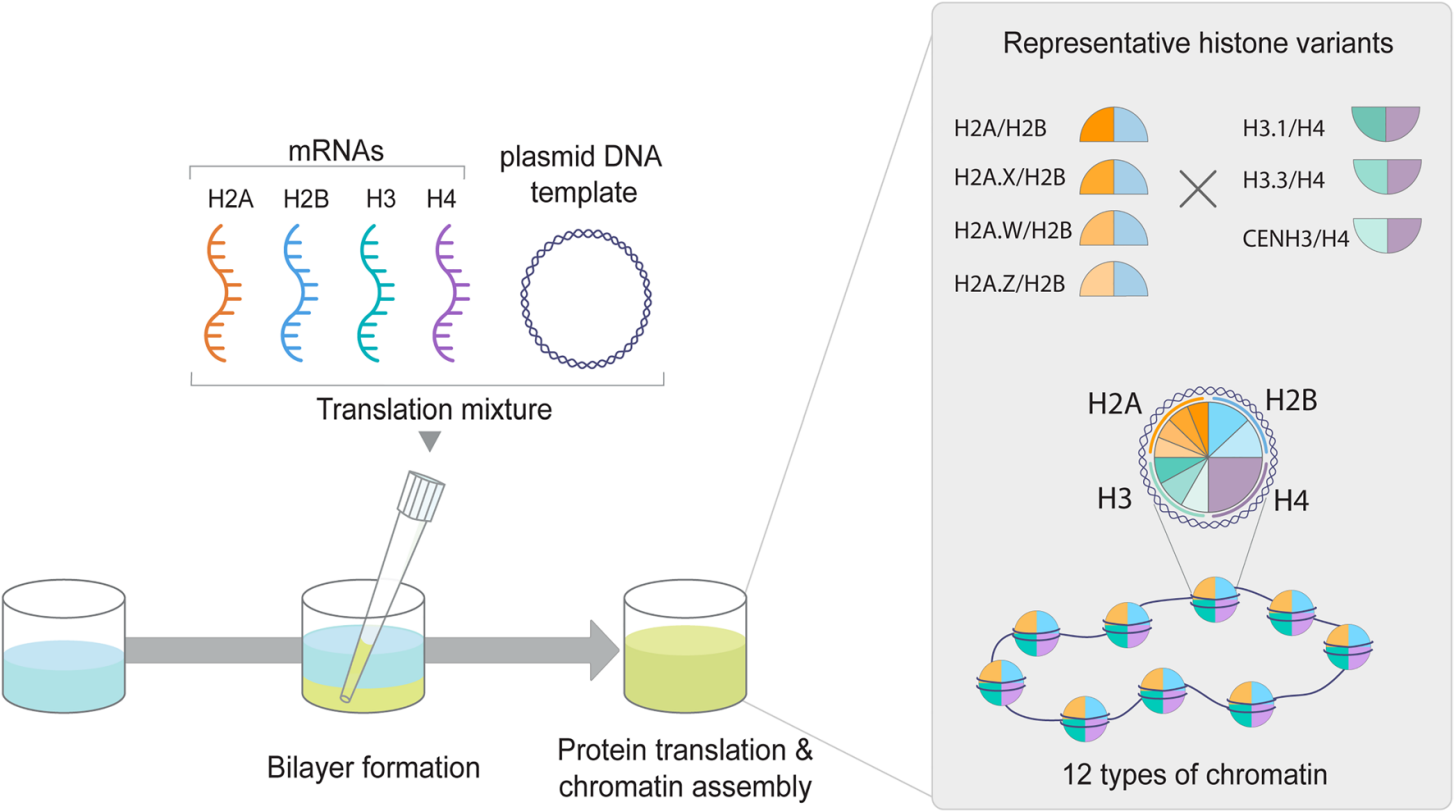
Schematic representation of this study. Schematic representation of the experimental setup for *A. thaliana* variant chromatin assembly using the wheat germ extract-based co-expression chromatin assembly platform.

**Figure 3:**
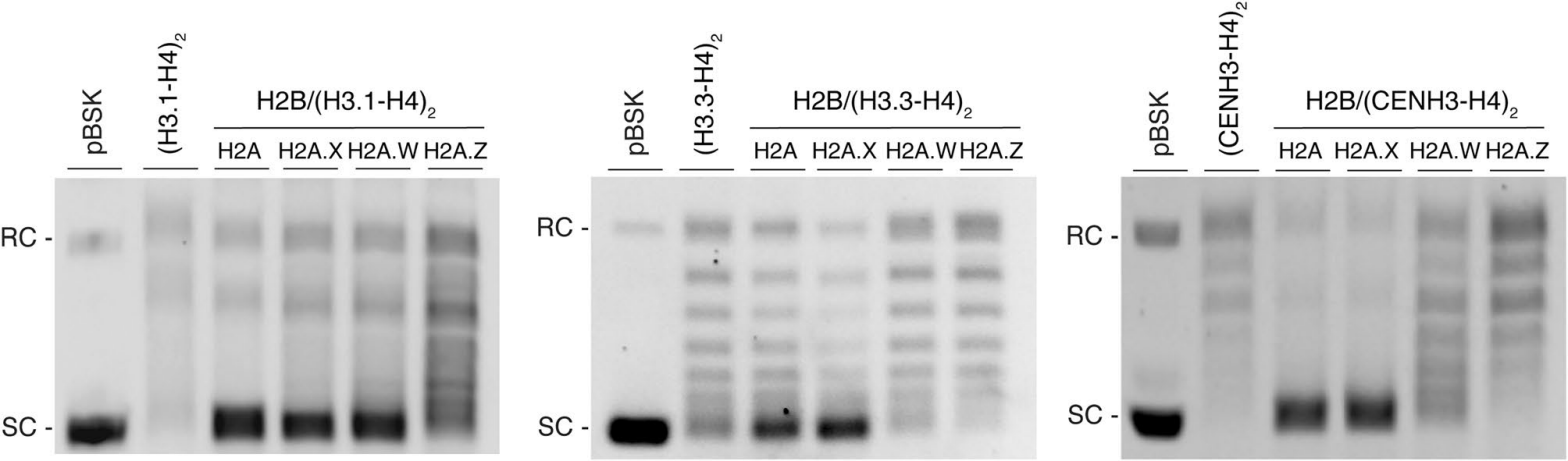
In vitro chromatin assembly of A. thaliana chromatin combinations. The supercoiling assay results of the reconstituted twelve nucleosome combinations were analyzed on a 0.8% 0.5xTBE agarose gel and visualized by EtBr. The first lane contains untreated, supercoiled pBSK plasmid, which serves as a reference plasmid DNA topology. The second lanes contain deproteinized pBSK plasmid from reaction mixtures that only contained (H3.1-H4)_2_, (H3.1-H4)_2_, or (CENH3-H4)_2_ tetrasome combinations. The chromatin combinations with four core histones are represented in the rest of the lanes. The SC and RC indicate the positions of the supercoiled and relaxed circular plasmid, respectively, estimated from the untreated, supercoiled pBSK plasmid.

To investigate if the wheat germ cell-free nucleosome assembly platform is suitable for the assembly of *A. thaliana* chromatin with histone variants, we co-expressed twelve combinations of canonical histones and their respective variants (Table 1) in the presence of relaxed pBSK plasmid DNAs^31–33^. Previously, a slight endogenous chromatin assembly activity was reported in the wheat germ extract during the reconstitution of *Drosophila* chromatin^33^. As both wheat and *Arabidopsis* are plants, we assumed *Arabidopsis* chromatin could be reconstituted in the wheat germ-based co-expression chromatin assembly system. The supercoiling assay was used for the evaluation of the assembly of twelve chromatin combinations. The chromatin assembly reaction was carried out accordingly to the previous studies^31–33^. Briefly, in vitro transcribed mRNAs coding for all four core histone proteins were mixed in optimized ratios and co-expressed in a single reaction in the presence of pBSK plasmid DNA and topoisomerase I (Fig. 2). The supercoiling assay was used for the evaluation of the assembly of chromatin containing histone variant combinations. In theory, the extent of DNA supercoiling is indicative of the degree of chromatin formation after deprotonation. Thus, the efficiency of chromatin assembly is evaluated by analyzing the proportion of relaxed and supercoiled plasmid DNA based on their migration patterns on an agarose gel^36^. In this assay, not only the chromatins with four core histones result in the formation of supercoiling, but also subnucleosomal species such as the H3-H4 tetrasome, an intermediate structure occurring during nucleosome assembly^37^. To distinguish supercoiling caused by subnucleosomal H3–H4 species or complete octameric nucleosome formation, the amounts of mRNAs encoding H3 and H4 were empirically set to a concentration, which does not exhibit complete supercoiling in a 4-h-long reaction (Fig. 3)^32^. Thus, the change in supercoiling in the presence of the respective H2A-H2B histones should represent the efficiency of octameric nucleosome formation. Out of twelve nucleosome core particle (NCP) combinations, seven NCPs, including H2A/H2B/H3.1/H4 (NCP), H2A.X/H2B/H3.1/H4 (H2A.X NCP), H2A.W/H2B/H3.1/H4 (H2A.W NCP), H2A/H2B/H3.3/H4 (H3.3 NCP), H2A.X/H2B/H3.3/H4 (H2A.X/H3.3 NCP), H2A/H2B/CENH3/H4 (CENH3 NCP), and H2A.X/H2B/CENH3/H4 (H2A.X/CENH3 NCP) showed apparent supercoiling formation relative to the respective H3-H4 controls, suggesting that this method suites to reconstitute *A. thaliana* chromatin with canonical histones and several histone variants (Fig. 3).

Regarding H2A and H2A.X-containing nucleosome combinations, that showed supercoiling regardless of the H3/H4 combinations, including H3.1/H4, H3.3/H4 and CENH3/H4, our observation is consistent with a previous in vivo genome-wide profiling study in *A. thaliana,* in which H2A.X was shown to be ubiquitously found throughout the genome with different types of histone H3s^8^. H2A.W showed substantial supercoiling in the presence of H3.1 but not with H3.3. Previous findings showed that H2A.W is strictly deposited in the heterochromatin regions and the pericentromeric regions with H3.1^8^, whereas H3.3 is reported to be enriched in euchromatin regions in *A. thaliana*^21,23^*.* Very recently, it was reported that H2A.W co-precipitated with both H3.1 and H3.3 in the same chromatin region by chromatin immunoprecipitation-seq (ChIP-seq), suggesting that H2A.W/H3.3 double variant nucleosomes are present in vivo^30^. Additionally, the combination of CENH3 and H2A.W, H2A.W/CENH3 NCP, was not supercoiled. Incompatibility between these variants may arise from the extended C-terminal tail of H2A.W that may be structurally unfavorable for CENH3 (Fig. 1c). None of the combinations containing H2A.Z were assembled in our system even when varied mRNA amounts were tested in the co-expression chromatin reconstitution reaction (S3), suggesting that H2A.Z might require specific factors for its deposition into chromatin, such as a histone chaperone^35,38^.

### Remodeling of histone variant-containing chromatin with a plant ISWI complex

**Figure 4:**
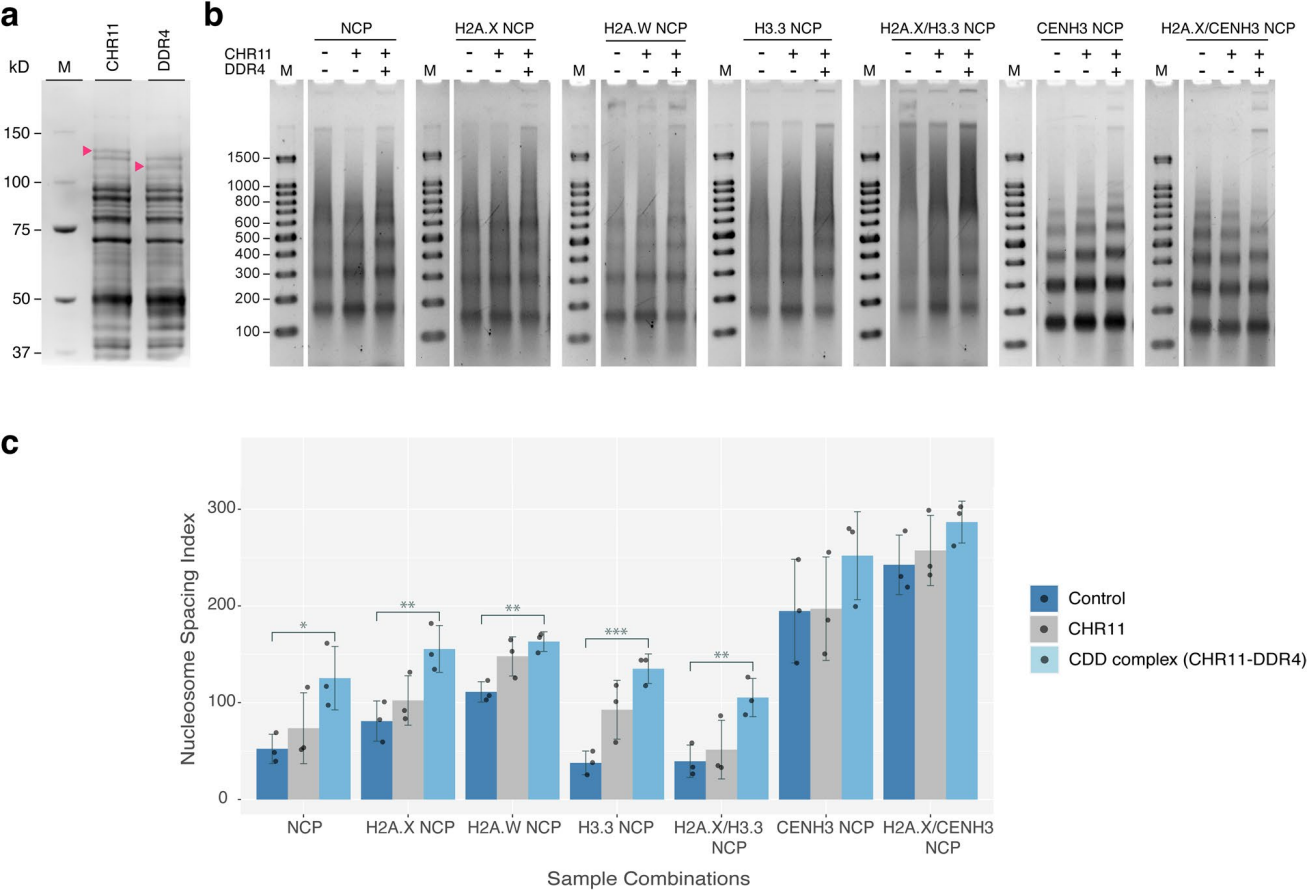
The effect of ISWI remodeling complex on seven reconstituted chromatin combinations. (**a**) The cell-free synthesized CHR11 and DDR4 proteins are shown on a 10% SDS-PAGE, stained by CBB. (**b**) The partial MNase digestion assay of the seven chromatin combinations (NCP, H2A.X NCP, H2A.W NCP, H3.3 NCP, H2A.X/H3.3 NCP, CENH3 NCP, and H2A.X/CENH3 NCP) reacted in the absence of remodeling factors, the presence of CHR11 mRNA or the presence of both CHR11 and DDR4 mRNAs were analyzed on a 2% agarose gel. Uncropped agarose gel images and technical replicates for (**b**) are available in Supplementary Information (S4–S6). (**c**) Nucleosome spacing indexes (NSIs) of each combination were estimated from the agarose gel densitometry results of MNase assay (n = 3), and mean NSI values were plotted on a bar graph. The jitter plots over the bar graphs show the individual values of three replicas, the individual graphs of each replicate can be found in the Supplementary Information (S9). Error bars represent the standard deviation from the three independent experiments. Asterisks denote the statistical significance of NSI values between samples: *p value < 0.05; **p value < 0.01; ***p value < 0.001; non-significant results were not plotted.

The seven reconstituted combinations were further tested as substrates for chromatin remodeling activity. Two imitation switch (ISWI) ATPase chromatin remodelers, CHR11 and CHR17, are known to facilitate nucleosome sliding and act redundantly to each other in *A. thaliana*^39,40^. In recent studies, these ISWI-type chromatin remodelers were reported to form complexes with single or multiple accessory proteins from the DDT-domain protein family^34,41^. Accessory proteins in the DDT-related (DDR) subfamilies, including DDR1, DDR3, DDR4, and DDR5, were reported to form a heterodimer with either CHR11 or CHR17^34^. Among DDRs, DDR4 was highly enriched when CHR11 or CHR17 was used as bait for immunoprecipitation followed by mass spectrometry in the *A. thaliana* cell lysate^35^. However, to the best of our knowledge, there is no direct evidence of whether the DDR4 functions with ISWI-type chromatin remodelers. Hence, we chose CHR11 and DDR4 for the remodeling assay for the seven successfully reconstituted chromatins. The CHR11 and DDR4 proteins were confirmed to be synthesized by the wheat germ cell-free reaction with their expected molecular weights (Fig. 4a). We co-expressed either CHR11 or CHR11/DDR4 (CDD complex) with seven types of variant-containing nucleosomes followed by partial Micrococcal Nuclease (MNase) assay. Partial MNase digestion generates an MNase ladder corresponding to mono-, di- and oligo nucleosomal DNAs. The deproteinized DNA digestion fragments were analyzed on agarose gel (Fig. 4b, and S4–S7). The nucleosome repeat lengths (NRLs) were estimated for the reconstituted chromatins in the absence or presence of remodeling activity from the MNase ladder corresponding to di-nucleosomes (n = 4 for the control samples and n = 3 for CDD complex containing samples). In the absence of CHR11 and DDR4 mRNAs, the average NRLs ranged from ~ 145 bp to 160 bp among canonical and six variant histone containing chromatins (Table 2 and S8), wherein all combinations in one gel image are supplemented separately (S7). The canonical (NCP) and H2A.X-containing (H2A.X NCP) chromatins showed NRLs of ~ 147 and ~ 149 bp, respectively, while the H2A.W NCP showed longer NRLs of ~ 158 bp, consistent with the previous report that H2A.W protects a longer stretch of DNA through its C-terminal KPSKK motif^5^. In H3.3-containing two chromatin combinations (H3.3 NCP and H2A.X/H3.3 NCP), apparent NRLs were ~ 149 bp. In contrast, the estimated NRLs of CENH3 NCP and H2A.X/CENH3 NCP of ~ 148 bp and 144 bp, respectively, were slightly shorter than other variants. In humans, CENP-A nucleosomes were found to have looser contact with the DNA at the entry-exit site^24^, resulting in high MNase sensitivity^42^, which could explain the observed shorter NRLs in two CENH3-containing chromatins.

**Table 2:** Effects of the CDD remodeling complex on the nucleosome repeat lengths of seven nucleosome types.

Nucleosome combinations	No remodeling activity (bp)*^1^	Presence of CDD complex (bp)*^2^
NCP	147 ± 3.4	151 ± 3.6
H2A.X NCP	149 ± 3.0	152 ± 2.2
H2A.W NCP	158 ± 3.2	161 ± 4.3
H3.3 NCP	150 ± 6.8	153 ± 3.9
H2A.X/H3.3 NCP	147 ± 6.9	152 ± 3.9
CENH3 NCP	148 ± 2.3	154 ± 0.7
H2A.X/CENH3 NCP	144 ± 1.3	150 ± 4

*^1^Average and standard deviation was estimated from 4 experimental replicas.

*^2^Average and standard deviation was estimated from 3 experimental replicas.

The NRLs of all nucleosome combinations appear to be longer when CHR11 or both CHR11 and DDR4 proteins (CDD complex) were co-expressed in the chromatin assembly reaction, indicating the remodeling function of CDD complex (Fig. 4b, Table 2 and S8). Additionally, compared to the control reaction, the CENH3-containing two nucleosomes, CENH3 NCP and H2A.X/CENH3 NCP, showed apparently longer NRLs of ~ 7 bp in the presence of the CDD complex. The longer NRLs found in the remodeled chromatins are consistent with the reported "ruler" function of the ISWI remodeling complex, which defines equal linker distances between neighboring nucleosomes in the chromatin context^43,44^. Our results further suggest that the length of the "ruler" might differ depending on the existing histone variants in chromatins.

To gain further insight into the remodeling function that would provide regular linker distances among the nucleosomes in the remodeled chromatins, the nucleosome spacing index (NSI) was estimated from the MNase results^45^ (Fig. 4b). Irregularly spaced nucleosomes will generate a pool of DNA fragments with random lengths, which appears as a background behind the MNase ladder or a smear. The NSI evaluates the periodicity of nucleosomes, by the intensity of the nucleosome bands and the background signal^45^. The NSI values were estimated from the agarose gel densitometric images of three replicas of each chromatin combination in the presence or absence of remodelers (S9). The mean and standard deviation of three independent replicas of each reaction condition were plotted on a bar graph (Fig. 4c). The nucleosome assembly reaction lacking remodeling activity (-CHR11/-DDR4) exhibited variance between the seven types of nucleosome combinations with an especially high spacing index for the two centromeric nucleosome combinations. The addition of CHR11 did not result in a statistically significant change in the values of nucleosome spacing except for H3.3 NCP (*p* > 0.05) (S10). When both CHR11 and the DDR4 accessory protein were co-expressed in the nucleosome assembly reaction, the nucleosome spacing index significantly increased in five combinations including NCP, H2A.X NCP, H2A.W NCP, H3.3 NCP, H2A.X/H3.3 NCP. The spacing activity was most pronounced for H3.3 NCP and H2A.X/H3.3 NCP, which were 2 to 2.5-fold greater than the control reaction (S10). These results suggest that the CDD remodeling complex indeed defines the regular spacing in the remodeled chromatin combinations. In contrast, the effect of CDD complex on NSIs was negligible in the CENH3-containing combinations, CENH3 NCP and H2A.X/CENH3 NCP. The little to no change in the NSIs in both CENH3-containing chromatins is likely attributed to the well-defined MNase ladders, even in the absence of the remodeling activity. To note, the remodeling activity observed when only CHR11 was co-expressed in our system was considerably lower compared to the values of estimated NRLs and NSIs in the presence of DDR4, except for H3.3 NCP. Thus, CHR11 needs an accessory protein to efficiently remodel chromatins (Fig. 4). Furthermore, these results suggest that the remodeling complex consisting of CHR11 and DDR4 provides different NRLs and regular spacings depending on the types of chromatins containing different histone variants. Our findings support the notion that ISWI-type remodelers can act preferentially toward variant-containing nucleosomes^46^.

## Conclusions

In the present study, we investigated the chromatin assembly properties of the wheat germ-based chromatin assembly platform for assembling various histone variant-containing nucleosome combinations from *A. thaliana*. We tested the assembly of canonical histones, H2A, H2B, H3.1, and H4, and histone variants, including three H2A variants and two H3 variants, resulting in twelve combinations of nucleosomes. Seven combinations, including the canonical NCP, were successfully reconstituted in a co-expression manner using the wheat germ cell-free chromatin reconstitution system and subjected to remodeling by co-expressing CHR11 and DDR4 proteins.

Overall, the current method is a useful tool to assemble nucleosomes with various histone variants, and to functionally determine the remodeling activities related to chromatin structure and physiological functions in plants.

## Methods

### Gene amplification by PCR using cDNA library

All targets for histones, remodeling factors, and histone chaperone were amplified from *A. thaliana* cDNA libraries using reverse and forward primers (Fasmac, Atsugi, Japan) with overhangs designed for ligation independent cloning, listed in Supplementary Table 1^47^. PCR products were purified by PEG precipitation (26% (w·v^−1^) PEG 8000, 6.5 mM MgCl_2_, 0.6 M sodium acetate, pH 7), followed by ethanol precipitation.

### Ligation independent cloning

Target genes were cloned into pEU0-E01-LICNot vector—designed for ligation independent cloning^47^. Briefly, the pEU0-E01-LICNot vector was linearized by SspI restriction enzyme and purified by gel extraction (Kanto Chemicals, Tokyo, Japan). Overhangs were created on both PCR products and the vector by T4 polymerase (Toyobo, Osaka, Japan), in the presence of dCTP and dGTP. The vector and the inserts were incubated at room temperature for 20 min in a 1:3 to 1:5 molar ratio, then transformed into *Escherichia coli* JM109 (Takara, Shiga, Japan). The clones were verified by DNA sequencing (Eurofins, Tokyo, Japan).

### In vitro transcription

The mRNA transcription reaction was conducted accordingly to the given protocol (CellFree Sciences, Yokohama, Japan). Briefly, 2 μg of plasmid constructs were transcribed by SP6 polymerase in the presence of ribonuclease inhibitor (Promega, Madison, WI) in a final volume of 20 μL reaction mixture for 4 h at 37 °C. 0.5 μL of 12 U·μL^−1^ DNase I (Nippon Gene, Tokyo, Japan) was added and incubated for 30 min at 37 °C, followed by acidic Phenol:Chloroform:Isoamyl alcohol (25:24:1, v·v^−1^) extraction and ethanol precipitation. The mRNA was resuspended in 10 μL nuclease-free water, and concentrations were measured by Denovix DS11 (Denovix, Wilmington, Delaware). Individually transcribed and purified mRNAs were mixed for the co-expression and chromatin reaction as described below^32^.

### Cell-free expression of individual proteins

Wheat germ extract (WEPRO7240H, CellFree Sciences) was used for the translation reaction of all histones and chromatin factors in a bilayer reaction mode, accordingly to the protocol provided by the company. Briefly, 10 μL of ~ 1 μg·μL^−1^ mRNA transcript solution, 10 μL wheat germ extract and 0.8 μL 1 mg·mL^−1^ creatine kinase were mixed and carefully layered under 206 μL of 1xSUB-AMIX (CellFree Sciences) to create a bilayer in a sterile microplate well. The translation reaction was performed for 20 h at 26 °C. The translated proteins were analyzed by an 18% SDS-PAGE, visualized by CBB staining. For western blot assay, the crude translation mixtures of the individually synthesized histone were separated on an 18% SDS-PAGE gel and transferred to a PVDF membrane (Biorad). The membrane was washed by TBS (Tris-Buffered Saline) buffer and blocked by EveryBlot blocking buffer (Biorad). The membrane was incubated with the respective polyclonal rabbit anti-histone antibodies at room temperature for 1 h or overnight at 4 °C in the recommended dilutions. Due to limitations in the availability of H2A variant-specific antibodies, two antibodies were used to detect canonical H2A and H2A variants: the canonical H2A was probed with anti-Histone H2A antibody II (#2578, Cell Signaling Technology, Danvers, Massachusetts, USA) at a dilution of 1:1000, and the H2A.X and H2A.W variants were detected with anti-H2A acidic patch antibody (Active motif, Carlsbad, California US) at a dilution of 1:1000. For the remaining histones the following antibodies were used: anti-H2B antibody (Abcam, Cambridge, United Kingdom, ab1790) at a dilution of 1:1000, anti-H3 antibody (ab1791) at a dilution of 1:1000, anti-CENH3 antibody (ab72001) at a dilution of 1:1000 dilution, anti-H4 (Active motif, Catalog No.: 61299) at a dilution of 1:500. Goat anti-rabbit IgG (H + L)-HRP conjugate (Biorad) was used as the secondary antibody at a dilution of 1:3000 and blots were developed using enhanced chemiluminescent reagents (Biorad). The membrane was visualized by LuminographII instrument (Atto, Tokyo, Japan).

### Co-expression chromatin assembly

Chromatin assembly grade wheat germ extract (WEPRO7240Ch, CellFree Sciences) was used to co-express all core histone combinations using approximately 2 μg each histone mRNA^31^. As a chromatin assembly control, 2 μg each of canonical H3 or variant H3 and H4-coding mRNA was used for the reaction. Briefly, individually transcribed and purified mRNAs were used to make 5 μL premix mRNA solutions for each chromatin combination and were mixed with 5 μL wheat germ extract, 0.4 μL of 1 mg·mL^−1^ creatine kinase, 0.5 μL of 0.5 μg·μL^−1^ pBSK plasmid, 0.1 μL of 20 U·μL^−1^ Topoisomerase I (Takara, Shiga, Japan), and adjusted with nuclease-free water to 10.7 μL. Topoisomerase I was added in order to release torsional strain during chromatin assembly onto circular closed plasmid. The reaction mixture was carefully layered under 103 μL of 1xSUB-AMIX (CellFree Sciences) to create a bilayer in a sterile microplate well. The chromatin assembly reaction was performed for 4 h at 26 °C.

### Supercoiling assay

50 μL translation mixture was digested by 1 μL of 600 U·mL^−1^ Proteinase K (Wako, Osaka, Japan), and the plasmid DNA was purified by Phenol:Chloroform:Isoamyl Alcohol (25:24:1, v·v^−1^, pH 8), followed by ethanol precipitation, and resolved in 6.5 μL HD buffer (25 mM HEPES, 1 mM DTT, pH 7.6) with the addition of a trace amount of Ribonuclease A (Macherey–Nagel GmbH & Co., Dueren, Germany). Samples were run on a 0.8% agarose gel in 0.5 × TBE buffer and stained by ethidium bromide. The gel image was analyzed using the ImageLab Software (Version 6.1.0 build 7, Biorad, Hercules, California).

### Micrococcal Nuclease Assay (MNase assay)

100 μL translation mixture was supplemented with 2.5 mM CaCl_2_ (final concentration) and digested by 0.1 U·μL^−1^ MNase (Takara, Shiga, Japan) at 37 °C. 35 μL aliquots were taken at 1 and 3 min and the reaction was halted by adding 5 mM EGTA. The DNA was purified as described above, and suspended in 4.5 μL HD buffer containing a trace amount of Ribonuclease A. The samples were analyzed on 2% agarose gel in 0.5 × TBE buffer and visualized by SAFELOOK™ Red Nucleic Acid Stain (Wako, Osaka, Japan). The gel image was analyzed using the ImageLab Software (Biorad). The nucleosome spacing indexes were estimated according to the previous study from the band intensity values of stained agarose gels^45^. Briefly, the spacing index was calculated by the following equation: (P3-P2)/2-V2, where P2 and P3 stand for the maximum intensity of densitometry peaks corresponding to the DNA digestion fragments of di- and trinucleosomes, respectively, and V2 stand for the minimum background intensity in the valley between P2 and P3 peaks^45^.

### Nucleosome remodeling

The co-translational nucleosome assembly was conducted as described above, with the addition of ~ 6 μg CHR11 (RefSeq number: NM_111515.5) either with or without ~ 6 μg DDR4 (RefSeq number: NM_001332380.1) mRNAs. Due to the increase in the number of proteins that need to be co-expressed with the four histones, the assembly reaction was performed for 16 h at 26 °C. The translation mix was subjected to Micrococcal Nuclease assay. Obtained NSIs from three independent replicas were statistically validated by the T-test function using R Studio (2023.03.1 + 446).

### Supplementary Information

Supplementary Information 1.

Supplementary Information 2.

## Data Availability

The experimental data generated or used during this study are either included in the article and the supplemental information.

## Abbreviations

NCP: Nucleosome core particle
NRLs: Nucleosome repeat lengths
NSI: Nucleosome spacing index
CHR11: CHROMATIN REMODELING11
DDR4: DDT domain-containing protein 4
CDD: CHR11-DDR4 complex
