# Supplementary material for: In vitro co-expression chromatin assembly and remodeling platform for plant histone variants
Source: Sci Rep. 2024 Jan 10;14:936. doi: 10.1038/s41598-024-51460-6 (PMC10776871; doi:10.1038/s41598-024-51460-6)
Supplement: Supplementary file 1 — Supplementary Information 1. [file 41598_2024_51460_MOESM1_ESM.docx]

**Supplementary data**

***In vitro* co-expression chromatin assembly and remodeling platform for plant histone variants**

Petra Banko^1,5^, Kei-ichi Okimune^1,2,5^, Szilvia K. Nagy^1,3^, Akinori Hamasaki^4^, Ryo Morishita^4^, Hitoshi Onouchi^1^, Taichi E. Takasuka*^1,2^

1 Graduate School of Agriculture, Hokkaido University, 060-0817 Sapporo, Japan

2 Graduate School of Global Food Resources, Hokkaido University, 060-0809 Sapporo, Japan

3 Department of Molecular Biology, Institute of Biochemistry and Molecular Biology, Semmelweis University, 1094 Budapest, Hungary

4 CellFree Sciences Co., Ltd, 790-8577 Matsuyama, Japan

5 These authors contributed equally: Petra Banko and Kei-ichi Okimune.

**Correspondence**

T. E. Takasuka, Graduate School of Agriculture, Hokkaido University, 060-0817 Sapporo, Japan

**Supplementary Methods**

**Proteomic analysis of H2A.Z.**

The *in vitro* synthesized histone H2A.Z was analyzed by an in-gel digestion LC-MS/MS using a modified protocol [1,2]. Briefly, 7 μL of H2A.Z in the translation mixture was run on an 18% SDS-PAGE gel and stained with CBB. The band corresponding to the expected size of H2A.Z was sliced to 1×1 mm pieces, and destained by soaking in 300 μL of 25 mM ammonium bicarbonate, 50% methanol, followed by incubation with 300 μL of 25 mM ammonium bicarbonate, 50% acetonitrile for 30 min at 40˚C. The gel piece was subjected to a reduction-alkylation step by incubating with 50 μL of 10 mM DTT, 25 mM ammonium bicarbonate for 45 min at 56˚C, then gently shaken with 50 μL of 55 mM iodoacetamide, 25 mM ammonium bicarbonate at room temperature for 30 min in the dark. 1.5 µL of 20 ng/µL Arg-C (Promega) was added to the reduced alkylated gel and incubated overnight at 37˚C. Peptides were extracted by adding 50 μL of 50% acetonitrile, 0.1% formic acid, and sonicated for 10 minutes then further extracted by adding 100% acetonitrile, 0.1% formic acid, and 100% H_2_O, 0.1% formic acid. The extracted peptides were purified by ZipTip (Millipore).

Proteomics analysis was performed using an Easy nLC1000 liquid chromatography system with Q-exacative plus Orbitrap mass spectrometer (Thermo Fisher Scientific) and Xcalibur software v. 3.1 (Thermo Fisher Scientific) for in-gel digested sample. The peptides were separated on a C18 capillary column (NTCC-360/75-3-125; Nikkyo Techno, Japan) by a linear gradient from 0 to 40% with two solutions; Solution A, 0.1% formic acid in water; Solution B, 0.1% formic acid in acetonitrile, for 120 min at a flow rate of 300 nL/min. Fullscan mass spectra were obtained in the mass spectrometer with a scan range of 300.0 to 2,000.0 m/z a resolution of 70,000 with maximum injection time of 50 ms and AGC 3e^6^. Proteome identification and quantification were performed from the acquired MS/MS spectra using Proteome Discoverer v. 2.1 (Thermo Fisher Scientific) with the *Arabidopsis* histone H2A.Z coding amino acid sequence. The precursor mass tolerance was set to 10 ppm and fragment mass tolerance was set to 0.6 Da for the proteomic analysis.

**References:**

1. Shevchenko, A., Wilm, M., Vorm, O., & Mann, M. Techniques in Protein Chemistry V. In *Mass Spectrometry in the Biological Sciences* **258**, 5 (1982).
2. Yohannes, E., Chang, J., Christ, G. J., Davies, K. P., & Chance, M. R. Proteomics analysis identifies molecular targets related to diabetes mellitus-associated bladder dysfunction. *Molecular and Cellular Proteomics* ***7***, 1270–1285. (2008).

| Primer name | Sequence |
| --- | --- |
| HTA10 forward | 5'-TACTTCCAATCCAATGCAATGGCGGGTCGTGGTAAAAC-3' |
| HTA10 reverse | 5'-TTATCCACTTCCAATGTCAATCGTCTTCAGCAGATGGCT-3' |
| HTA3 forward | 5'-TACTTCCAATCCAATGCAATGAGTTCCGGCGCCGG-3' |
| HTA3 reverse | 5'-TTATCCACTTCCAATGTTAAAACTCTTGAGAAGCAGATCCGATATCG-3' |
| HTA6 forward | 5'-TACTTCCAATCCAATGCAATGGAATCCACCGGAAAAGTG-3' |
| HTA6 reverse | 5'-TTATCCACTTCCAATGTTAAGCTTTCTTTGGAGACTTGACTG-3' |
| HTA9 forward | 5'-TACTTCCAATCCAATGCAATGTCGGGGAAAGGTGCTA-3' |
| HTA9 reverse | 5'-TTATCCACTTCCAATGCTATTCCTTGGCGGATTTGTTG-3' |
| HTB9 forward | 5'-TACTTCCAATCCAATGCAATGGCGCCGAGAGCAGAGAA-3' |
| HTB9 reverse | 5'-TTATCCACTTCCAATGTCAAGAGCTTGTGAATTTGGTAACAGCC-3' |
| HTR2 forward | 5'-TACTTCCAATCCAATGCAATGGCTCG5'-TACCAAGCAG-3' |
| HTR2 reverse | 5'-TTATCCACTTCCAATGCTAAGCTCGTTCTCCTCTG-3' |
| HTR5 forward | 5'-TACTTCCAATCCAATGCAATGGCTCG5'-TACTAAGCAAACAG-3' |
| HTR5 reverse | 5'-TTATCCACTTCCAATGTTAAGCACGTTCTCCTCTGATC-3' |
| HTR12 forward | 5'-TACTTCCAATCCAATGCAATGGCGAGAACCAAGCATC-3' |
| HTR12 reverse | 5'-TTATCCACTTCCAATGTCACCATGGTCTGCCTTTTC-3' |
| HIS4 forward | 5'-TACTTCCAATCCAATGCAATGTCAGGAAGAGGAAAAG-3' |
| HIS4 reverse | 5'-TTATCCACTTCCAATGTCAACCACCAAATCCATATA-3' |
| CHR11 forward | 5'-TACTTCCAATCCAATGCAATGGCGAGAAATTCGAATTCC-3' |
| CHR11 reverse | 5'-TTATCCACTTCCAATGTCATCTCATCGACAGGTGC-3' |
| DDR4 forward | 5'-TACTTCCAATCCAATGCAATGGGTTCCTCCTCCGA-3' |
| DDR4 reverse | 5'-TTATCCACTTCCAATGTTATGAATTGTCTGTCTTATCATCA-3' |
| NAP1;3 forward | 5'-TACTTCCAATCCAATGCAATGAGCAACGATAAGGACAG-3' |
| NAP1;3 reverse | 5'-TTATCCACTTCCAATGTTACTGTTGTTTGCATTCAGG-3' |

**Supplementary Table 1. Primers designed for ligation-independent cloning.**

**
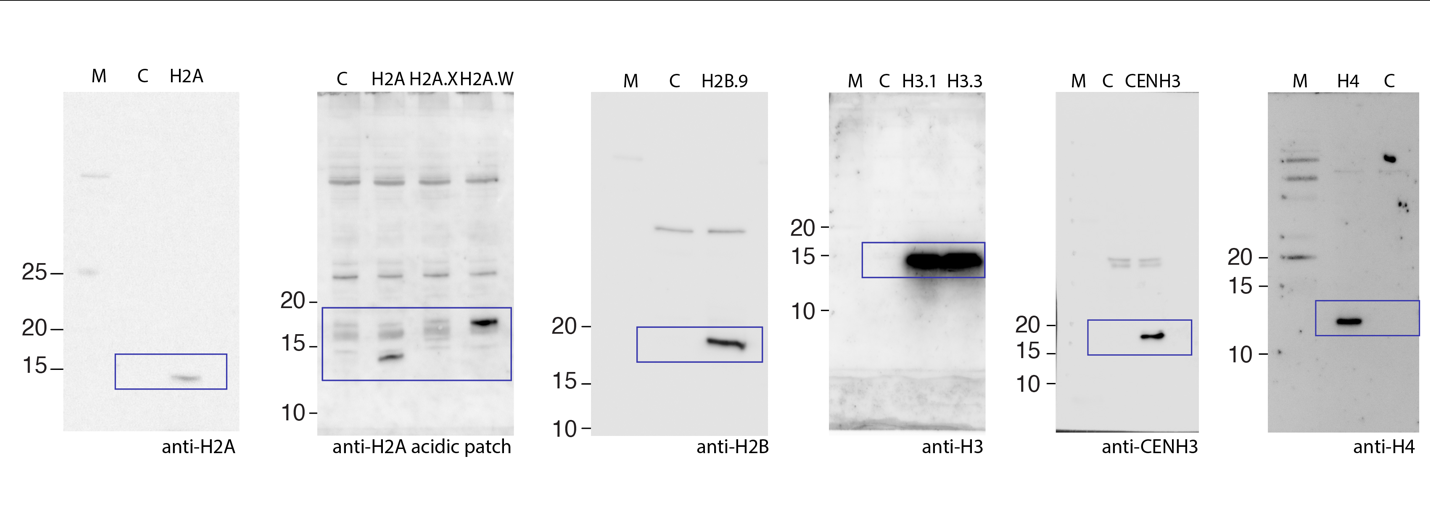
**

**Supplementary Figure 1. Uncropped Western blots from Figure 1b.** Blue boxes indicate the areas which were used for Fig. 1b.


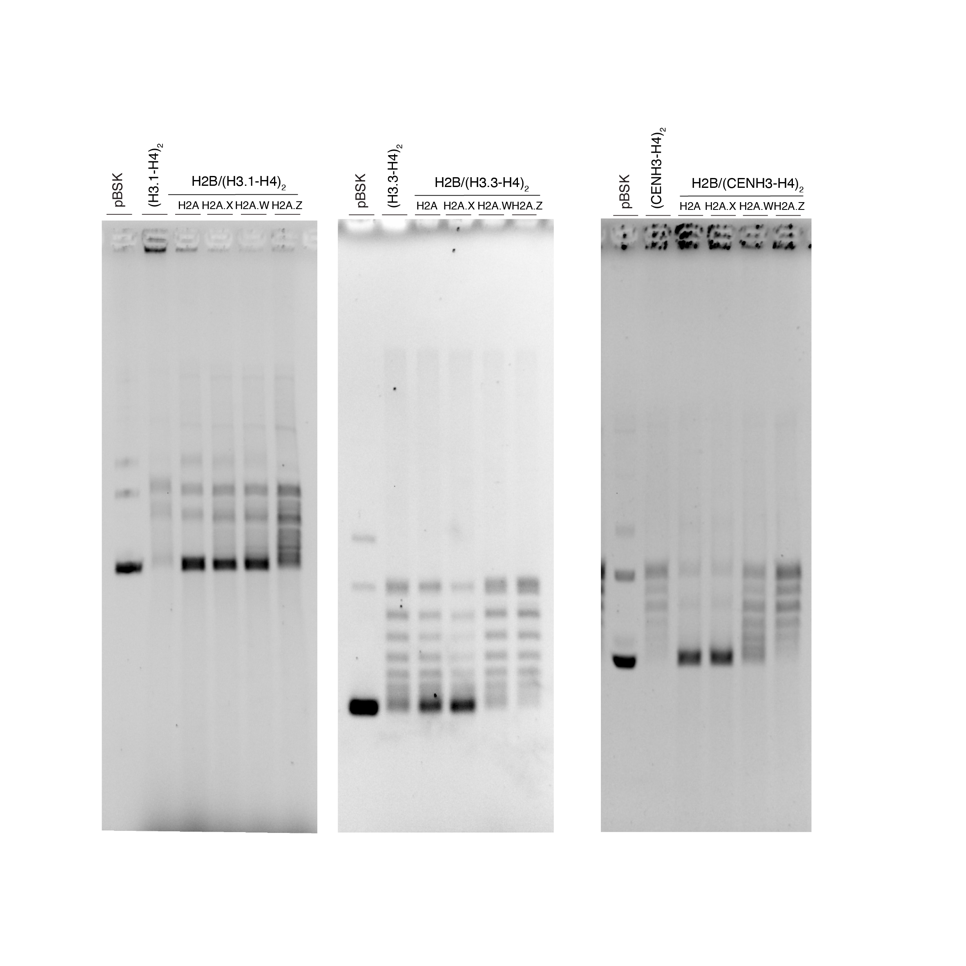


**Supplementary Figure 2. Uncropped DNA supercoiling assays gels of *Arabidopsis* chromatin combinations of Figure 3.**

**
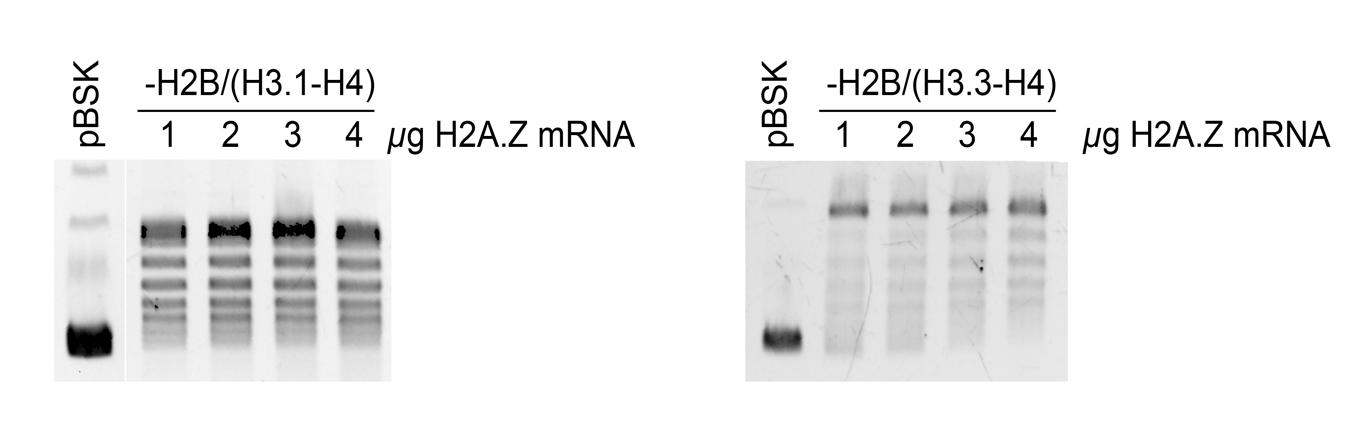
**

**Supplementary Figure 3. Supercoiling assay results from different amounts of H2A.Z mRNA input in the reconstitution reactions. Two chromatin assembly reactions containing H2A.Z were tested with the range of 1 to 4 µg H2A.Z mRNA in the series of reactions. The amount of the H2B, the respective H3 variants, and H4 mRNAs were fixed at the same concentration.**


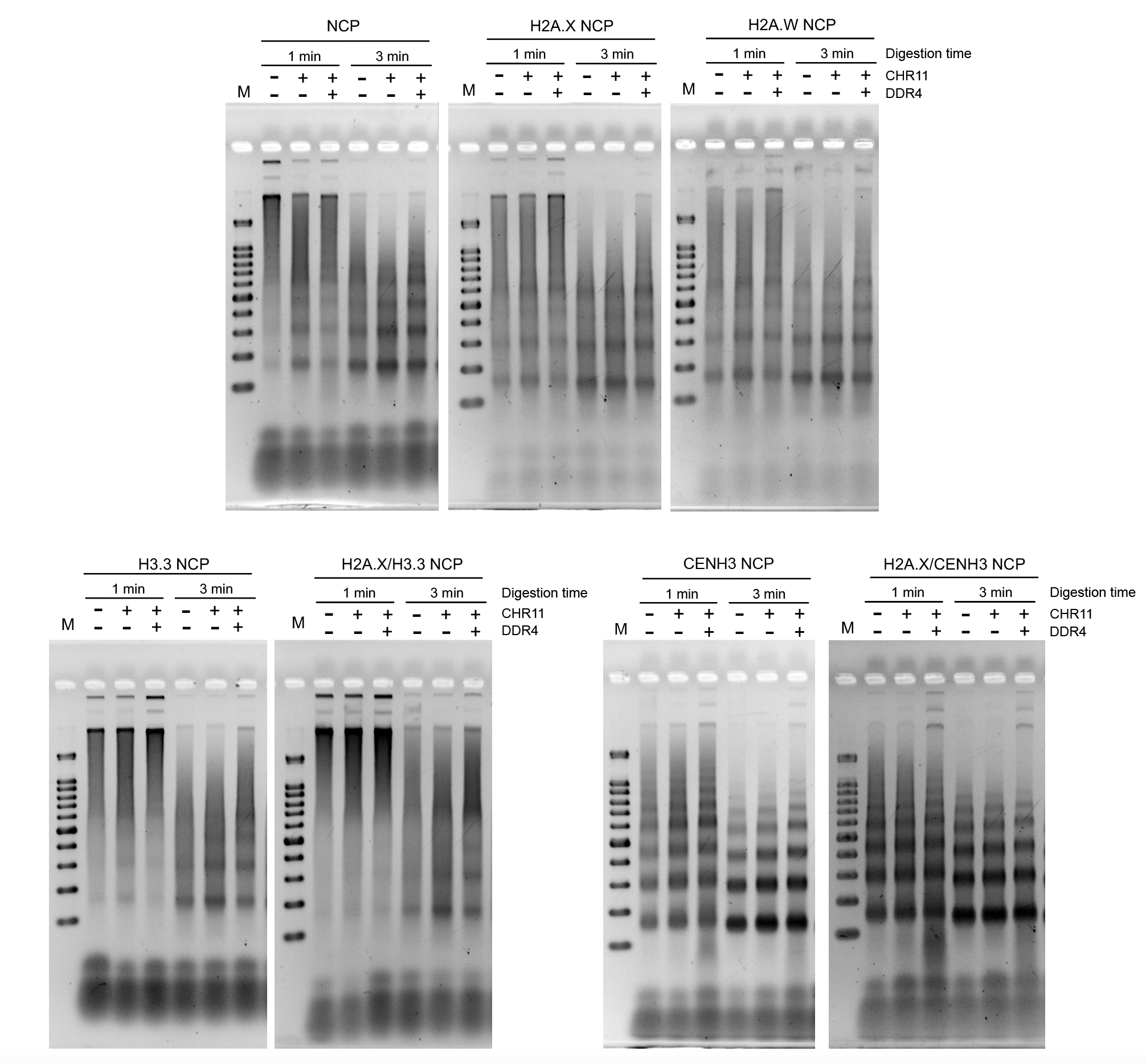


**Supplementary Figure 4. Uncropped partial MNase assay results of Figure 4.** Each sample contained the same concentrations of MNase enzyme, and the reactions were stopped at 1 and 3 minutes. Deproteinized DNA samples were separated on a 2% agarose gel and visualized by GelRed.

**
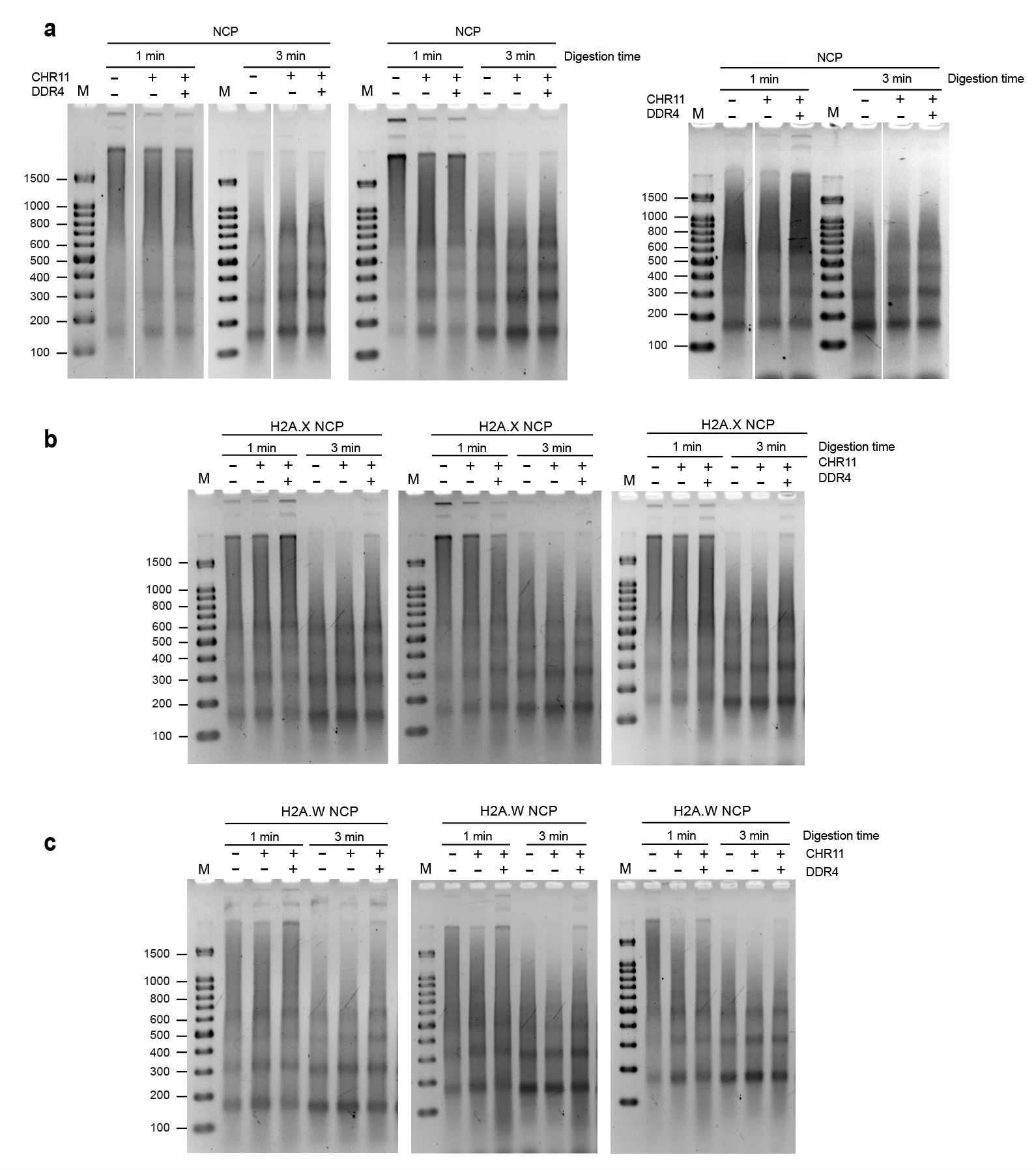
**

**Supplementary Figure 5. Technical replicas of partial MNase assay results of Figure 4.**

Each sample contained the same concentrations of MNase enzyme. The reactions were stopped at 1 and 3 minutes, deproteinized samples were separated on a 2% agarose gel and visualized by GelRed.

**
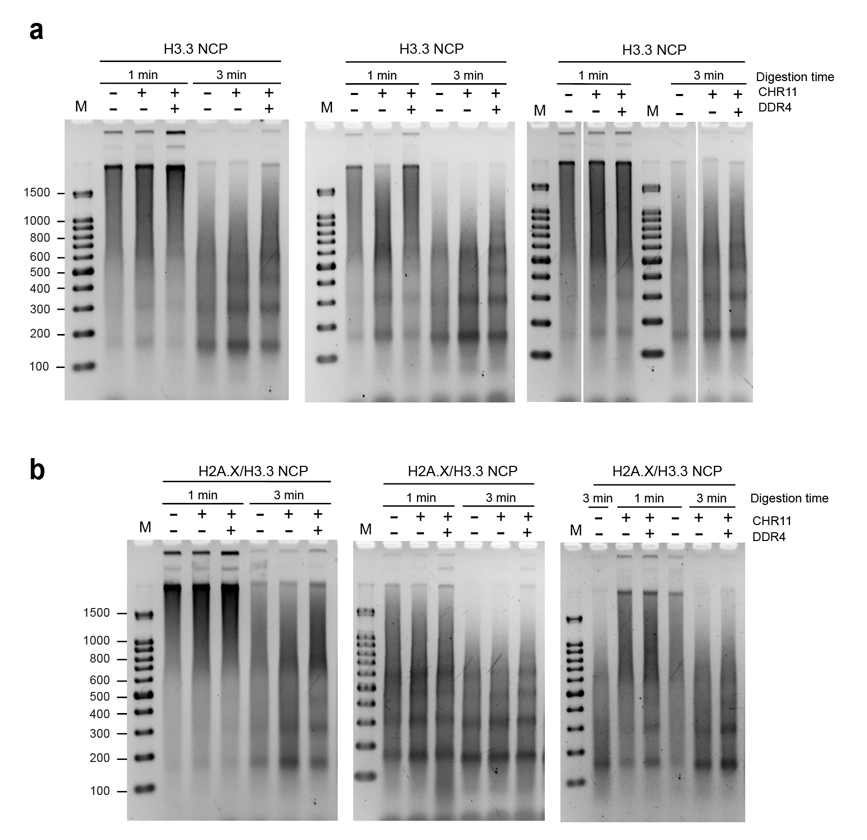

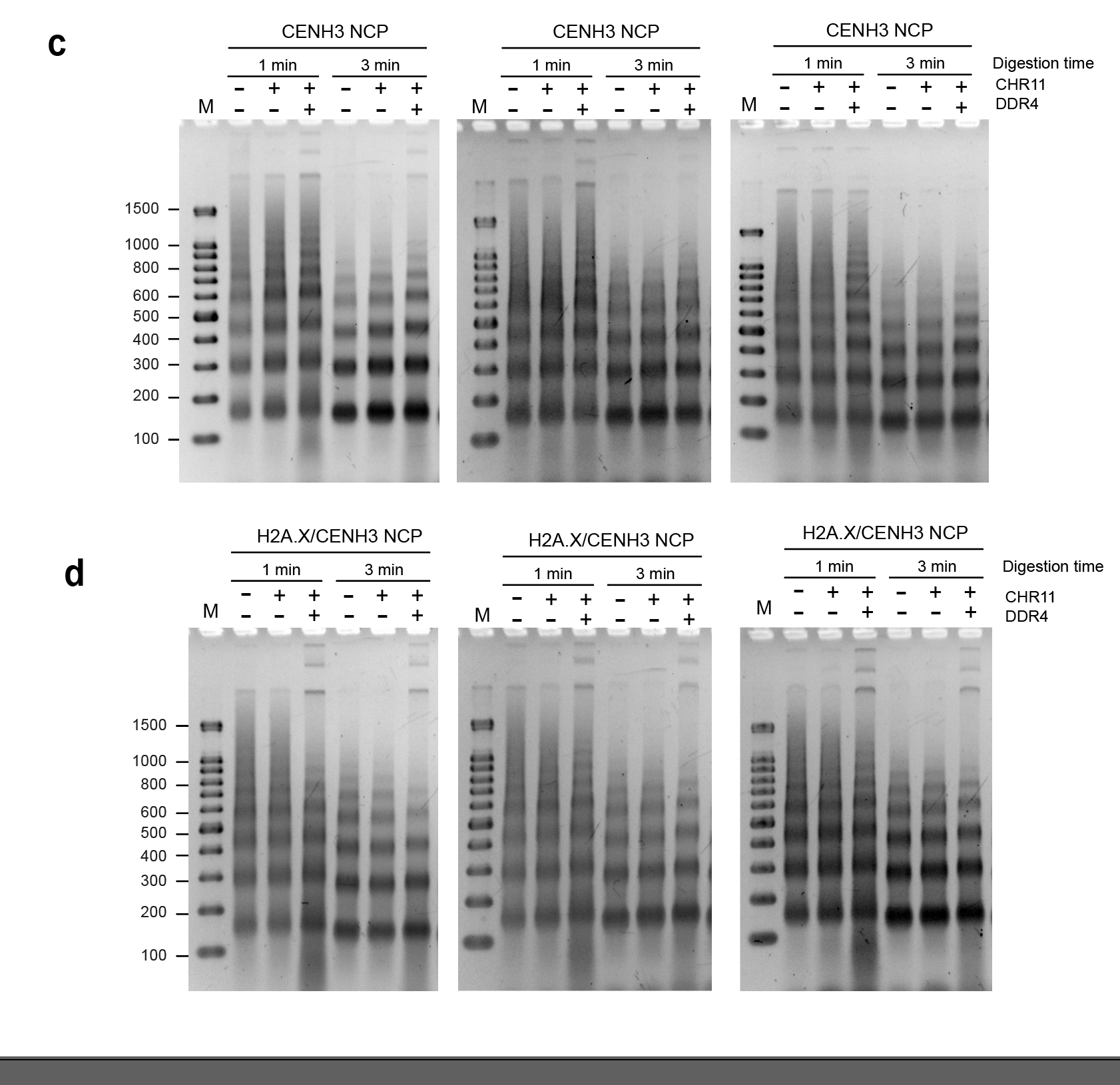
**

**Supplementary Figure 6. Technical replicas of partial MNase assay results of Figure 4. H3.3 and CENH3 nucleosome combinations.** Each sample contained the same concentrations of MNase enzyme, and the reactions were halted at 1 and 3 minutes and deproteinized samples were separated on a 2% agarose gel GelRed staining.


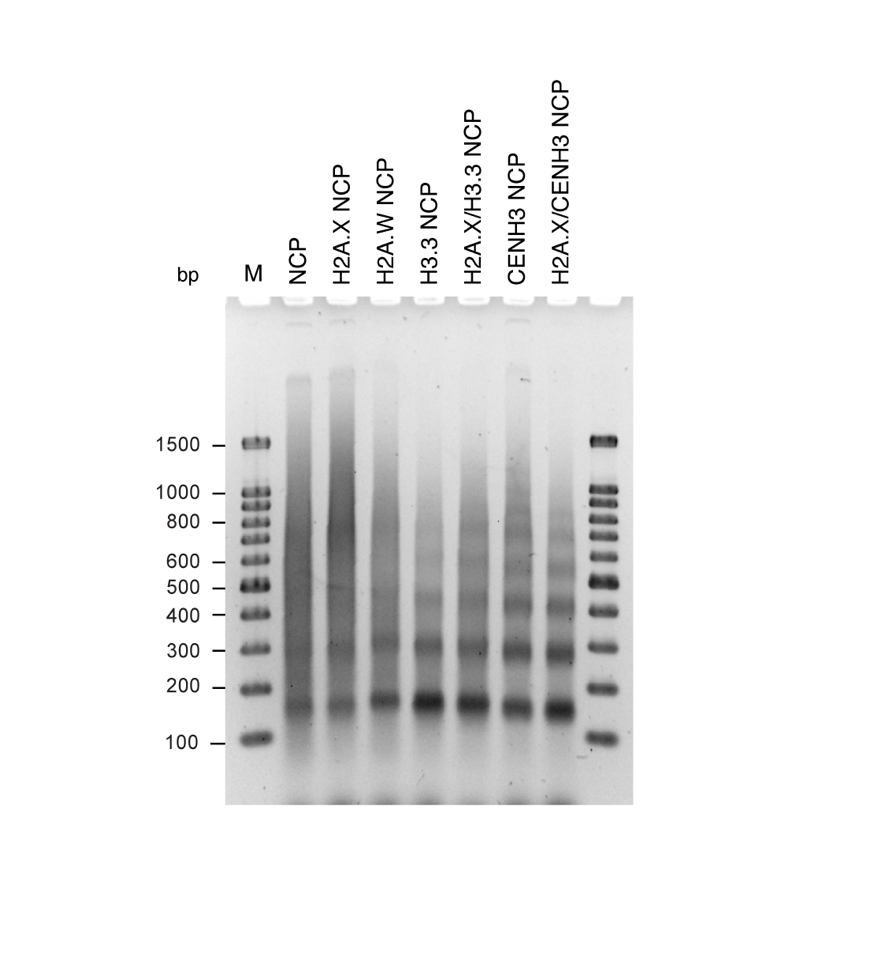


**Supplementary Figure 7. MNase assay results of the seven chromatin combinations without remodelers.** The seven chromatin combinations (NCP, H2A.X NCP, H2A.W NCP, H3.3 NCP, H2A.X/H3.3 NCP, CENH3 NCP, and H2A.X/CENH3 NCP) were assembled in a 16-hour reaction and digested by MNase for 3 minutes. The extracted DNA fragments were separated on a 2% agarose gel and stained with GelRed.

**Supplementary Figure 8. The effects of the CDD remodeling complex on the nucleosome repeat lengths (NRLs) of seven nucleosome types. The NRLs were determined by analyzing the MNase images obtained from three separate reactions. The error bars represent the standard deviation of technical replicates** (n=4 for the control samples and n=3 for CDD complex-containing samples).

**Supplementary Figure 9. The effect of ISWI remodeling complex on seven reconstituted chromatin combinations. Three technical replicas are shown in their separate bar graphs from Fig. 4c. The graph depicts the calculated NSIs of the seven types of chromatin combinations in the presence or absence of remodelers (CHR11, CDD).**

**
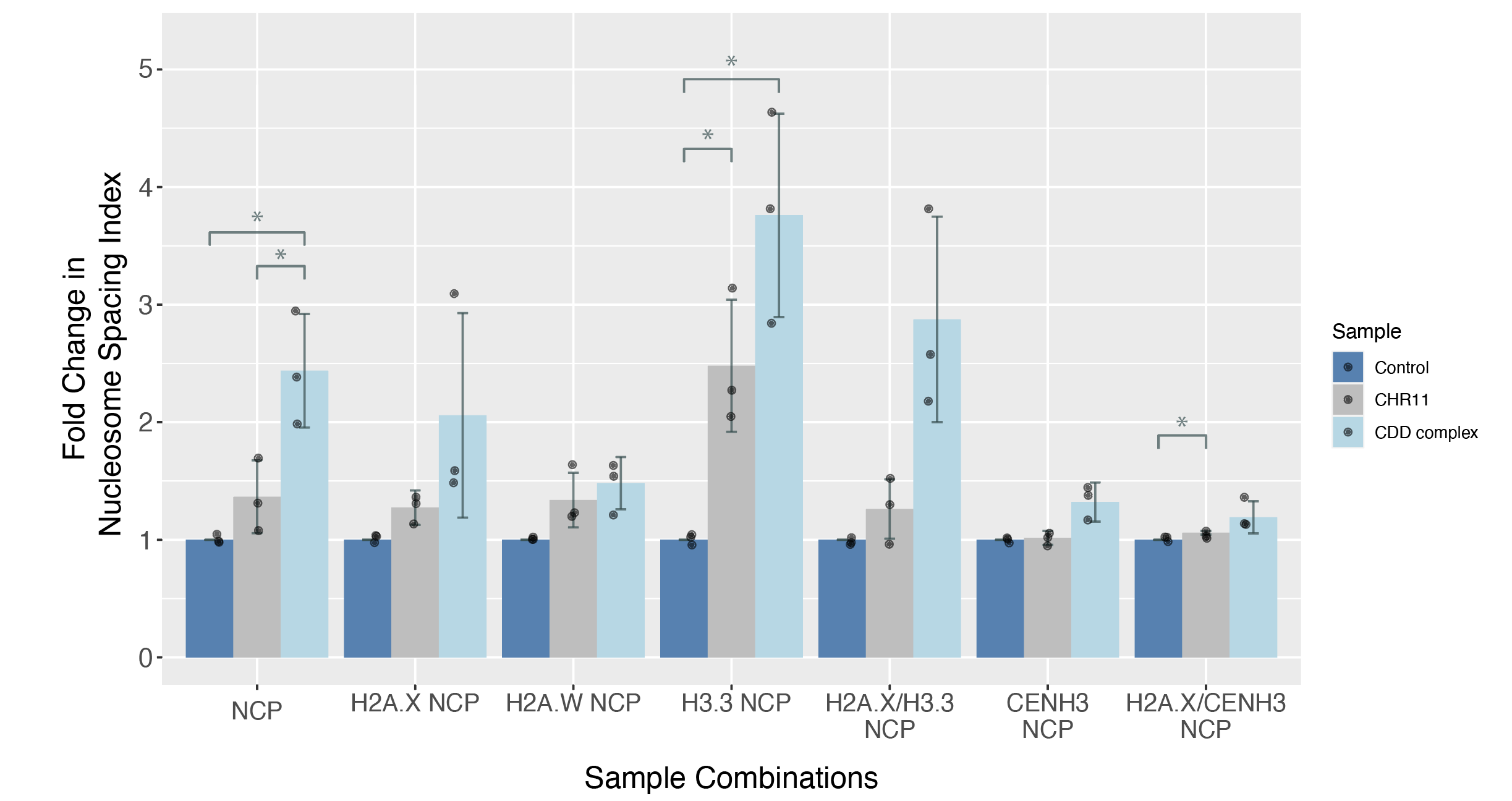
**

**Supplementary Figure 10. The effect of ISWI remodeling complex on seven reconstituted chromatin combinations relative to the control reaction without remodelers. To express the effect of remodelers on nucleosome spacing (Fig. 4c), we calculated the fold change in nucleosome spacing indexes and plotted them on a bar graph. The bar height shows the mean values of three technical replicates and the** error bars indicate the standard deviation. The jitter plots over the bar graphs show the fold change of three replicas. Error bars represent the standard deviation. Asterisks denote the statistical significance of fold change in NSIs: *p-value< 0.05; **p-value <0.01; ***p-value <0.001; non-significant results were not plotted.
